# Reproducibility and validity of food group intake in a short food frequency questionnaire for the middle-aged Japanese population

**DOI:** 10.1186/s12199-021-00951-3

**Published:** 2021-03-02

**Authors:** Nahomi Imaeda, Chiho Goto, Tae Sasakabe, Haruo Mikami, Isao Oze, Akihiro Hosono, Mariko Naito, Naoko Miyagawa, Etsuko Ozaki, Hiroaki Ikezaki, Hinako Nanri, Noriko T. Nakahata, Sakurako K. Kamano, Kiyonori Kuriki, Yuri T. Yaguchi, Takamasa Kayama, Ayako Kurihara, Sei Harada, Kenji Wakai

**Affiliations:** 1 Department of Nutrition, Faculty of Wellness, Shigakkan University, Obu, Aichi Japan; 2 Department of Public Health, Nagoya City University Graduate School of Medical Sciences, Nagoya, Aichi Japan; 3 Department of Health and Nutrition, School of Health and Human Life, Nagoya Bunri University, Nagoya, Aichi Japan; 4 Department of Public Health, Aichi Medical University, Nagakute, Aichi Japan; 5 Department of Preventive Medicine, Nagoya University Graduate School of Medicine, Nagoya, Aichi Japan; 6 Cancer Prevention Center, Chiba Cancer Center Research Institute, Chiba, Chiba Japan; 7 Division of Epidemiology and Prevention, Aichi Cancer Center Research Institute, Nagoya, Aichi Japan; 8 Department of Oral Epidemiology, Hiroshima University Graduate School of Biomedical and Health Sciences, Hiroshima, Hiroshima Japan; 9 Department of Public Health, Shiga University of Medical Science, Otsu, Shiga Japan; 10 International Center for Nutrition and Information, National Institutes of Biomedical Innovation, Health and Nutrition, Shinjuku, Tokyo Japan; 11 Department of Epidemiology for Community Health and Medicine, Kyoto Prefectural University of Medicine, Kyoto, Kyoto Japan; 12 Department of General Internal Medicine, Kyushu University Hospital, Fukuoka, Fukuoka Japan; 13 Department of Comprehensive General Internal Medicine, Kyushu University Faculty of Medical Sciences, Fukuoka, Fukuoka Japan; 14 Section of Behavioral Physiology, Department of Physical Activity Research, National Institutes of Health and Nutrition, National Institutes of Biomedical Innovation, Health and Nutrition, Shinjuku, Tokyo Japan; 15 Department of Health and Nutrition, Faculty of Nursing and Nutrition, University of Shimane, Hamada, Shimane Japan; 16 Department of Preventive Medicine, Tokushima University Graduate School of Biomedical Sciences, Tokushima, Tokushima Japan; 17 Laboratory of Public Health, Graduate School of Nutritional and Environmental Sciences, Shizuoka University, Shizuoka, Shizuoka Japan; 18 Department of Advanced Cancer Science, Faculty of Medicine, Yamagata University, Yamagata, Yamagata Japan; 19 Department of Preventive Medicine and Public Health, Keio University School of Medicine, Shinjuku, Tokyo Japan

**Keywords:** Dietary survey, Food frequency questionnaire, Validity, Reproducibility, Cohort study, Japanese

## Abstract

**Purpose:**

The purpose of this study was to evaluate the reproducibility and validity of a short food frequency questionnaire (FFQ) for food group intake in Japan, the reproducibility and partial validity of which were previously confirmed for nutrients.

**Methods:**

A total of 288 middle-aged healthy volunteers from 11 different areas of Japan provided nonconsecutive 3-day weighed dietary records (DRs) at 3-month intervals over four seasons. We evaluated reproducibility based on the first (FFQ1) and second (FFQ2) questionnaires and their validity against the DRs by comparing the intake of 20 food groups. Spearman’s rank correlation coefficients (SRs) were calculated between energy-adjusted intake from the FFQs and that from the DRs.

**Results:**

The intake of 20 food groups estimated from the two FFQs was mostly equivalent. The median energy-adjusted SRs between the FFQ1 and FFQ2 were 0.61 (range 0.38–0.86) for men and 0.66 (0.45–0.84) for women. For validity, the median de-attenuated SRs between DRs and the FFQ1 were 0.51 (0.17–0.76) for men and 0.47 (0.23–0.77) for women. Compared with the DRs, the proportion of cross-classification into exact plus adjacent quintiles with the FFQ1 ranged from 58 to 86% in men and from 57 to 86% in women. According to the robust Z scores and the Bland–Altman plot graphs, the underestimation errors in the FFQ1 tended to be greater in individuals with high mean levels of consumption for meat for men and for other vegetables for both men and women.

**Conclusion:**

The FFQ demonstrated high reproducibility and reasonable validity for food group intake. This questionnaire is short and remains appropriate for identifying associations between diet and health/disease among adults in Japan.

**Supplementary Information:**

The online version contains supplementary material available at 10.1186/s12199-021-00951-3.

## Introduction

In epidemiological studies for dietary factors, researchers have investigated the association between dietary intake and health outcomes [1]. For outcomes of lifestyle-related diseases, diet may affect the risk over a long period of time. Considering the great intra-individual variations, it is important to estimate an individual’s habitual dietary intake instead of short-term intake. As a dietary assessment tool, the food frequency questionnaire (FFQ) has often been used because it can capture usual dietary intake among free-living people. Although the FFQ is relatively easy to answer, questionnaires with many items may pose a challenge to responders. According to a previous review, the validity of the FFQ increases only slightly even if it contains more than 100 items [2]. Therefore, considering the cost and burden on the respondents, many researchers tend to prefer the use of short-form FFQs.

A shorter 47-item version of the FFQ developed by Tokudome et al. has been shown to have reasonable validity for estimating nutrient intake [3]. This version of the FFQ has been widely used throughout Japan because of its brevity [4–6], including in large-scale cohort studies [7–9]. We examined and confirmed the validity of the 47-item FFQ based on 3-day weighed dietary records (DRs) [10, 11] and assessed its reproducibility [12], but only in the central area of Japan (Aichi Prefecture); therefore, whether its validity and reproducibility are generalizable to all of Japan remains unclear. Furthermore, the FFQ has not been validated for food group intake. In addition to nutrient intake, food group consumption should be assessed in relation to disease risk to obtain useful knowledge for prevention. Therefore, validation studies of FFQs at the food group level are also important.

With this background, the aim of this study was to assess the validity and reproducibility of a 47-item short FFQ for food group intake among middle-aged men and women in multiple cohorts.

## Methods

### Participants and study schedule

In our study, the sample size was determined to show that the FFQ is reasonably valid for nutrient intakes instead of food group consumption. This is because the primary measure is the validity for nutrient intakes; the validity for food group consumption is a secondary measure. The sample size (*n* = 285) was calculated so that the Pearson correlation coefficients between nutrient intake as estimated by the FFQ and those derived from DRs would be significantly different from 0.30 based on the assumptions of a coefficient of 0.45 [2] (*a* = 0.05 and *b* = 0.20) and dropout rate of 10% (*n* = 28–30) [13].

We recruited 308 individuals from 9 areas in the Japan Multi-Institutional Collaborative Cohort Study (J-MICC study), Yamagata Molecular Epidemiological Cohort, and Tsuruoka Metabolomics Cohort to participate in a dietary survey from September 2011 to October 2013. The details regarding the study areas and the number, age, and sex of the participants enrolled from each of the study areas are summarized in Table 1. To be eligible for participation in the present study, the respondents had to be 35–69 years of age at baseline and live in the study area of each cohort.

**Table 1:** Dietary survey protocol for each area and the participants’ characteristics

Area	DR							FFQ1		FFQ2		Included in final analyses^§^		
			Baseline	Completed	Excluded	Within criteria		*n*		*n*		Age, mean	BMI, mean (SD)	
	Start year	Methods	All	All	All	Men	Women	Men	Women	Men	Women	All	Men	Women
Yamagata	2013	Note, photos	28	28	1	12	15	12	15	12	15	54.5	23.7 (2.6)	23.0 (4.3)
Tsuruoka	2013	Note, photos	32	32	0	17	15	17	15	17	15	48.5	24.8 (4.5)	22.2 (3.3)
Chiba	2013	Note, photos	28	28	0	14	14	14	14	14	13	54.1	23.6 (4.4)	21.2 (2.9)
Shizuoka-Sakuragaoka^#^	2013	Note, photos	27	27	1	13	13	13	13	13	11	46.1	22.3 (1.9)	21.0 (3.1)
Shizuoka	2012	Note, photos	26	26	0	13	13	13	13	13	13	52.6	24.4 (2.5)	20.8 (2.6)
Okazaki	2013	Note, photos	39	39	1	17	21	17	21	17	20	48.8	23.1 (2.8)	21.3 (3.3)
Aichi Cancer Center	2012	Note, photos	29	29	0	15	14	15	14	15	14	51.3	23.2 (2.8)	22.7 (4.7)
Takashima	2012	Note, photos	24	23	0	12	11	12	11	12	11	48.5	22.8 (2.9)	21.9 (2.4)
Kyoto	2013	Note, photos	22	16	0	6	10	6	10	5	9	50.1	25.3 (3.1)	21.5 (3.4)
Tokushima	2012	Note, photos	29	28	2	14	12	14	12	14	12	52.9	22.6 (2.8)	22.0 (1.9)
Saga	2011	Note, photos	24	24	0	12	12	12	12	11	12	55.6	22.8 (2.5)	23.3 (4.0)
Total			308	300	5	145	150	145	150	143	145	51.4	23.4 (3.1)	21.9 (3.4)

*BMI* body mass index, *DR* four nonconsecutive 3-day weighed dietary records at intervals of 3 months (12 days), *FFQ*: 47-item short food frequency questionnaire, *SD* standard deviation

§ The age and BMI are those at baseline among participants included in the final analyses (men = 143, women = 145)

# The numbers of dropouts in the Shizuoka-Sakuragaoka area was unknown

Eight participants failed to complete the DRs owing to their busy schedule and other difficulties. Of the remaining 300 individuals, we further excluded one who retracted her consent, three who recorded their diet for more than 12 days, and one who did not fill in both the first (FFQ1) and the second (FFQ2) questionnaires. In addition, seven participants skipped either the FFQ1 or FFQ2. Eventually, 143 men and 145 women who completed the 12-day DR, the FFQ1, and the FFQ2 were included in the analysis for reproducibility and validity. The response rate was not calculated because we did not record the number of individuals who were asked to participate in this dietary survey.

In the validation study, we collected data on the participants’ age, sex, height, weight, and physical activity level using a self-administered questionnaire at the beginning of the survey. The validation study was scheduled as illustrated in Additional file 1. The participants fulfilled the first FFQ1. Four 3-day DRs (DR1–DR4) were then conducted at 3-month intervals, and the respondents were asked to answer the FFQ2 at 2 months after the last DR. The responses to the FFQ2 were compared with those to the FFQ1 to assess reproducibility. The FFQ was validated by comparing the food group intake estimated by both the FFQ1 and FFQ2 with the intake derived from the DRs as a reference. Further details of the FFQs and DRs are described below.

### Dietary intake using the 47-item FFQ

The self-administered FFQ includes question items on the average frequency of consumption during the past year with the following eight possible responses: almost never, 1–3 times per month, 1–2 times per week, 3–4 times per week, 5–6 times per week, once per day, twice per day, and ≥ 3 times per day. These responses are then converted into intake scores of 0, 0.1, 0.2, 0.5, 0.8, 1, 2, and 3, respectively, to approximate the intake frequency per day. The consumption of foods is tabulated in grams for 20 food groups based on the Standard Tables of Food Composition in Japan (seventh revised edition) [14]. The food groups (shown as the number of items: description) were rice (1), bread (1), noodles (1), potatoes (1), soybean products (4: tofu in miso soup, tofu dishes, fermented soybeans [*nattō*], and fried tofu [*ganmodoki*]), green vegetables (5: pumpkin, carrot, broccoli, green-leaf vegetables, and other green-yellow vegetables), other vegetables (5: cabbage, radish, dried radish [*kiriboshi-daikon*], bamboo shoots, and other vegetables), fruit (2: citrus fruits and other fruits), mushrooms (1), seaweeds (1), fish (7: fish, bone-edible small fish, canned tuna, octopus/shrimp/crab, shellfish, fish eggs, and fish paste products), meat (4: chicken, beef/pork, liver, and ham/sausage/bacon), eggs (1), milk (2: milk and yogurt), oils (6: margarine, butter, mayonnaise, deep-fried dishes, light-fried dishes/sauté, and peanuts/almonds), confectionery (2: Western- and Japanese-style confectioneries), green tea (1), coffee (1), alcoholic beverages (1), and soybean paste (1).

The FFQ contains no question items on usual portion size for 43 food items, so we applied the standard portion sizes based on DRs in a population from Aichi Prefecture [3]. However, portion sizes are requested for three kinds of staple foods in Japan (rice, bread, and noodles). The daily consumption of each food item was computed by multiplying the portion size by the intake score. For alcoholic beverages, the amount and frequency per week or month were asked for the following 10 items: sake, Japanese liquor (*shōchū*), *shōchū* highball, large bottle of beer (633 mL), medium-sized bottle of beer (500 mL), 350 mL of canned beer, 250 mL of canned beer, single whiskey, double whiskey, and wine. Sugar-sweetened beverages (SSBs) were not included in the short FFQ.

### Reference method

Nonconsecutive 3-day DRs including one weekend day at 3-month intervals over four seasons, that is, 12-day DRs, were collected. Prior to the dietary survey, the participants were guided individually or in small groups on how to complete the DRs by their district dietitians. Participants were asked to record their intake of all foods, dishes, and drinks using a notebook and pictures. A digital scale with a maximum weighing capacity of 2 kg was recommended for measuring dishes with a large portion size (e.g., noodle and rice bowls). No special products were specified. Pictures were taken using checkered-pattern luncheon mats as a scale whenever possible. The managers of each study area were trained by research dietitians (N. I. and C. G.) and the explanations given to the study participants about the DRs were standardized between areas using a common DR manual. Pictures taken with mobile phones, smartphones, and digital cameras were not adopted as the gold standard, but were requested to help supplement the written records, obtain the product names of the confectionery and processed foods, and estimate the portion sizes and volumes. Since 2011, a research dietician (N.I.) has conducted quality control on weighing, recording, and taking photographs of meals in all areas [15–17]. Between 2012 and 2014, staff dietitians were monitored for compliance with the participants’ instructions. Compliance was rated on a 5-point scale, with “5” indicating almost complete compliance and “1” almost none; these scores were provided as feedback to the staff. The main items checked were the use of luncheon mats; taking pictures of breakfast, lunch, and dinner; taking pictures of snacks; and recording the names of the food ingredients, the amount of food in approximate amounts, and the weight of the food according to the food scales. For each season, the registered dietitians confirmed the details of the DRs by phone or email (or in an interview for the first DR in some cases) when the descriptions in the DRs were unclear. Data from all regions were retrieved using a standardized data checking algorithm, and suspicious data (such as outliers and missing seasoning data) were checked and corrected.

### Statistical analysis

Body mass index (BMI) was calculated using the following formula: body weight [kg] / (height [m])^2^. Energy consumption according to the FFQs and DRs was estimated using the Standard Tables of Food Composition in Japan (fifth edition) [18]. The reason for using the fifth edition was so that we could match the editions adopted in the development of the FFQ. We confirmed that the energy values of the foods appearing in the dietary survey in the fifth edition were identical to those in the seventh and most recent edition.

#### Intake in each food group

For food group intakes, the residual method was performed to adjust the energy intake [1]. In this method, the residuals were computed as those from the regression model with total energy intake as the independent variable and food group consumption as the dependent variable. The energy-adjusted food group consumption was calculated for each subject as the residual plus food group intake corresponding to the mean energy intake. We calculated means, standard deviations (SDs), medians, and interquartile ranges (IQRs) (25th to 75th percentiles) for the FFQ1, FFQ2, and DRs separately for men and women. We also assessed the differences between the estimated values by FFQs and those by DRs by using the following equation for a robust Z score [19, 20]:

$$ \mathrm{Robust}\ \mathrm{Z}-\mathrm{score}=\left(\mathrm{Dx}-\mathrm{Dref}\right)/\mathrm{NIQR} $$

where Dx is the median of evaluated dietary intake, Dref is the median of reference dietary intake, and NIQR is the normalized interquartile range.

The coefficient for converting the IQR to a normal distribution was 1.349 (NIQR = IQR/1.349). The absolute value of the robust Z score was regarded as acceptable if it was less than 0.5 for reproducibility and less than ± 1.0 for validity.

#### Correlation

We evaluated the reproducibility for each food group intake between the FFQ1 and FFQ2 using crude data, and energy-adjusted (adjusted by the residual method) Spearman’s rank correlation coefficients (SRs). Validity was evaluated using energy-adjusted SRs and energy-adjusted de-attenuated SRs between the FFQ1, FFQ2, and DRs. The energy-adjusted de-attenuated SRs indicate correlations adjusted for random intra-individual errors from the usual intake of each food group [1, 21]. The intra-individual variations between the four 3-day DRs were considered in this analysis. The correlation coefficients, calculated using the density method for energy adjustment, were also shown in Additional file 2.

#### Agreement

For reproducibility, we examined categorical agreement between the estimated intake on the FFQ1 and FFQ2. For validity, we examined categorical agreement between the calculated intake on both FFQs and in the DRs. We computed the number of participants classified into the same, adjacent, and extreme categories by cross-classification according to quintile.

#### Bland–Altman plot graphs

To check for systematic errors, Bland–Altman plot graphs showing markers of a healthy diet (rice, fish, meat, milk, other vegetables, and fruit) were drawn using the energy–adjusted intake from the FFQ1 and DR data (adjusted by the residual method) [22]. Illustrations of the Bland–Altman plot between the FFQs and DRs can explain systematic errors, namely fixed and proportional biases; the former is a type of error that tends to be consistent in magnitude and/or direction independently, while the latter proportionally increases with the values in the DR [23, 24]. This error may occur due to the over/underestimated portion size in the FFQ.

All analyses were performed using SPSS Statistics (version 25; IBM Japan, Tokyo, Japan).

## Ethical considerations

The study protocol, including the reuse of data collected before the study, was approved by the ethical review board of Aichi Cancer Center (No. 3-50, 2011) before the study began. The addition of participating institutions was also approved by the review board (No. 3-48, 2013) prior to the dietary survey conducted by those research groups. Written informed consent was obtained from the participants after they had received explanations about the study purpose and methods.

## Results

The baseline characteristics of the participants in this study are shown in Table 1. The mean ± SD of BMI was 23.4 ± 3.1 kg/m^2^ for men and 21.9 ± 3.4 kg/m^2^ for women.

### Intake of each food group

The median of daily energy-adjusted intake (adjusted by the residual method) of rice, which is a major energy contributor in the FFQ1, FFQ2, and DRs, were 453, 434, and 364 g for men and 272, 267, and 226 g for women, respectively (Table 2). There were no food groups with a robust Z score outside ± 0.5 between the FFQ1 and FFQ2 in men and women.

**Table 2:** Food group intakes (g/day) according to two FFQs, dietary records, and robust Z scores in men and women

	FFQ1				FFQ2				DR				Robust Z score		
Food group	Mean	(SD)	Median	IQR	Mean	(SD)	Median	IQR	Mean	(SD)	Median	IQR	FFQ1vs. FFQ2	DR vs. FFQ1	DR vs.FFQ2
Men															
Energy (kcal)	2295	496	2228	1994–2546	2301	481	2200	1952–2583	2253	359	2226	2021–2498	− 0.1	0.0	− 0.1
Rice	447	189	453	308–571	425	153	434	325–530	377	128	364	296–467	− 0.1	0.7	0.6
Bread	53	50	41	15–84	52	44	47	16–80	50	32	47	26–68	0.1	− 0.2	0.0
Noodles	49	37	44	29–58	51	40	43	28–59	92	55	81	52–122	− 0.1	− 0.7	− 0.7
Potatoes	16	12	11	9–24	15	16	12	8–17	45	21	41	31–58	0.1	− 1.5	− 1.5
Soybean products	64	37	54	38–79	62	40	54	36–86	74	56	61	42–86	0.0	− 0.2	− 0.2
Green vegetables	60	36	50	35–78	62	44	52	34–76	118	68	97	68–159	0.0	− 0.7	− 0.7
Other vegetables	54	31	48	32–66	52	32	47	30–60	190	75	173	142–217	0.0	− 2.3	− 2.3
Fruit	47	47	31	20–66	44	43	26	16–67	129	109	96	45–185	− 0.2	− 0.6	− 0.7
Mushrooms	7	5	5	3–10	6	5	5	3–8	14	12	12	7–19	0.0	− 0.7	− 0.7
Seaweeds	1	1	1	1–1	1	1	1	1–2	13	11	10	5–18	0.0	− 1.0	− 1.0
Fish	46	24	39	31–58	47	25	43	30–60	80	41	73	53–102	0.2	− 0.9	− 0.8
Meat	39	18	34	25–49	43	24	37	28–58	106	38	104	78–131	0.2	− 1.8	− 1.7
Eggs	20	13	19	9–33	20	15	20	8–32	44	20	44	29–57	0.0	− 1.2	− 1.2
Milk	99	91	73	29–153	108	104	88	22–161	106	93	71	37–161	0.2	0.0	0.2
Oils	15	7	14	9–19	16	9	16	10–21	27	8	27	21–32	0.2	− 1.6	− 1.4
Confectionery	20	20	18	8–26	19	20	15	9–24	27	25	21	12–34	− 0.2	− 0.2	− 0.4
Green tea	278	239	220	44–440	270	248	192	56–465	246	255	178	58–326	− 0.1	0.2	0.1
Coffee	142	103	101	51–202	153	107	118	65–216	143	160	93	20–203	0.2	0.1	0.2
Alcoholic beverages	137	169	80	17–238	146	173	82	21–207	187	220	135	38–255	0.0	− 0.3	− 0.3
Soybean paste	13	10	11	7–15	12	10	10	5–15	11	7	10	7–14	− 0.3	0.3	− 0.1
Median for *Z* score for food intakes													0.0	− 0.6	− 0.7
Women															
Energy (kcal)	1942	341	1935	1725–2092	1928	350	1890	1718–2105	1752	269	1720	1541–1902	− 0.2	0.8	0.6
Rice	273	98	272	205–358	260	92	267	183–329	234	79	226	180–295	0.0	0.5	0.5
Bread	46	37	43	14–70	48	37	44	20–70	49	26	48	32–64	0.0	− 0.2	− 0.2
Noodles	40	35	25	13–50	41	32	36	22–53	73	42	67	46–89	0.4	− 1.3	− 1.0
Potatoes	19	13	13	10–28	18	15	11	10–27	38	20	34	23–47	− 0.1	− 1.2	− 1.3
Soybean products	64	33	61	43–77	59	32	54	37–75	63	40	54	36–79	− 0.3	0.2	0.0
Green vegetables	89	53	79	53–108	82	50	72	50–99	108	47	100	73–136	− 0.2	− 0.4	− 0.6
Other vegetables	86	42	75	58–107	81	40	75	55–101	172	57	164	130–203	0.0	− 1.6	− 1.6
Fruit	64	48	53	24–89	59	45	49	25–84	117	76	105	63–153	− 0.1	− 0.8	− 0.8
Mushrooms	11	7	10	6–13	10	6	9	4–15	16	12	13	8–20	− 0.1	− 0.4	− 0.4
Seaweeds	2	2	1	1–3	2	1	1	1–2	11	9	9	5–14	− 0.2	− 1.1	− 1.2
Fish	46	24	43	29–55	43	20	42	29–52	62	29	57	42–80	0.0	− 0.5	− 0.5
Meat	45	21	42	31–58	44	17	41	32–57	73	26	70	54–93	0.0	− 1.0	− 1.0
Eggs	23	14	20	10–32	22	13	20	9–32	36	15	34	25–46	0.0	− 0.8	− 0.9
Milk	148	112	132	52–207	141	115	125	51–182	137	95	116	78–173	− 0.1	0.2	0.1
Oils	17	7	16	12–21	17	7	16	12–20	23	7	23	19–27	0.0	− 1.0	− 1.0
Confectionery	20	13	17	13– 24	23	17	20	13–29	37	23	32	23–47	0.4	− 0.9	− 0.7
Green tea	306	226	208	122–592	304	228	210	91–582	289	262	224	95–415	0.0	− 0.1	− 0.1
Coffee	155	106	170	79–216	155	106	192	78–210	128	123	90	24–203	0.2	0.6	0.8
Alcoholic beverages	42	98	8	0–49	42	106	13	− 2–51	72	136	32	1–77	0.1	− 0.4	− 0.3
Soybean paste	11	9	12	3–15	11	8	11	3–15	8	5	7	4–11	− 0.1	0.9	0.8
Median for *Z* score for food group intakes													0.0	− 0.4	− 0.5

The residual method was performed to adjust the energy intake. *FFQ* 47-item short food frequency questionnaire, *DR* four 3-day weighed dietary records at intervals of 3 months (12 days), *SD* standard deviation, *IQR* interquartile range

Robust Z score = (Dx − Dref)/NIQR; Dref: median in reference dietary intake, NIQR: normalized interquartile range = interquartile range/1.349, Dx: median in evaluating dietary intake, 1.349 is the coefficient for converting IQR to a normal distribution

When comparing the FFQs with the DRs, rice intakes on the FFQs were higher than those in the DRs for both men and women. In particular, robust Z scores below − 1.5 between the FFQ1 and DRs were found for other vegetables (− 2.3), meat (− 1.8), and oils (− 1.6) in men, and other vegetables (− 1.6) in women; robust Z scores between the FFQ2 and DRs showed similar differences. The lowest robust Z scores were found for other vegetables in both men and women. In men, the median of daily energy-adjusted intake of other vegetables was 173 g in the DRs and 48 g in the FFQ1, which was underestimated by 72%. Similarly, in women, the median intake of other vegetables in the FFQs was approximately 54% lower than that in the DRs.

### Correlation coefficient and agreement rate for reproducibility

Crude and energy-adjusted SRs by FFQ1 vs. FFQ2 are markers for the reproducibility for food group intake. In men, energy-adjusted SRs were distributed from 0.38 (seaweeds) to 0.86 (alcoholic beverages), with a median of 0.61 (Table 3). The percent of exact agreement between the FFQ1 and FFQ2 according to quintile categorization was 42% as the median, with a range from 31% (seaweeds) to 55% (alcoholic beverages) for men. The agreement for the same and adjacent category was 81% (range 70–94%) as the median for men (Table 4). Extreme disagreement rates (median) were 1% for men. In women, energy-adjusted SRs by FFQ1 vs. FFQ2 were distributed from 0.45 (seaweeds) to 0.84 (alcoholic beverages), with a median of 0.66 (Table 3). The agreement rate for women was 41% as the median, with a range from 32% (seaweeds) to 62% (coffee). Agreement for the same and adjacent category was 80% (range 69–92%) as the median (Table 4). Extreme disagreement rates (median) were 1% for women.

**Table 3:** Spearman's rank correlation coefficients for reproducibility and validity for men and women

			Men ( *n* = 143)						Women ( *n* = 145)			
	FFQ1 vs. FFQ2		FFQ1 vs. DR		FFQ2 vs. DR		FFQ1 vs. FFQ2		FFQ1 vs. DR		FFQ2 vs. DR	
	Crude	Energy-adjusted	Energy-adjusted	De-attenuated	Energy-adjusted	De-attenuated	Crude	Energy-adjusted	Energy-adjusted	De-attenuated	Energy-adjusted	De-attenuated
Rice	0.78	0.70	0.63	0.67	0.60	0.63	0.83	0.74	0.56	0.61	0.62	0.66
Bread	0.70	0.67	0.69	0.76	0.69	0.75	0.82	0.75	0.62	0.70	0.62	0.70
Noodles	0.59	0.50	0.48	0.61	0.52	0.65	0.71	0.70	0.27	0.36	0.46	0.63
Potatoes	0.43	0.40	0.11	0.17	0.17	0.26	0.60	0.59	0.29	0.41	0.25	0.34
Soybean products	0.75	0.67	0.30	0.36	0.32	0.37	0.71	0.70	0.40	0.47	0.37	0.44
Green vegetables	0.63	0.57	0.31	0.34	0.32	0.35	0.66	0.64	0.31	0.36	0.42	0.49
Other vegetables	0.50	0.46	0.31	0.35	0.27	0.30	0.62	0.57	0.21	0.24	0.21	0.24
Fruit	0.76	0.73	0.57	0.62	0.63	0.69	0.70	0.65	0.51	0.58	0.42	0.48
Mushrooms	0.58	0.49	0.21	0.26	0.28	0.34	0.57	0.56	0.20	0.26	0.26	0.33
Seaweeds	0.46	0.38	0.28	0.35	0.25	0.30	0.45	0.45	0.17	0.23	0.25	0.33
Fish	0.67	0.61	0.32	0.38	0.53	0.61	0.72	0.67	0.49	0.58	0.58	0.69
Meat	0.56	0.53	0.36	0.41	0.37	0.42	0.63	0.61	0.36	0.41	0.37	0.43
Eggs	0.71	0.70	0.42	0.48	0.43	0.50	0.69	0.67	0.38	0.47	0.43	0.52
Milk	0.77	0.72	0.71	0.76	0.76	0.81	0.86	0.82	0.52	0.56	0.51	0.54
Oils	0.54	0.56	0.33	0.38	0.33	0.39	0.65	0.60	0.29	0.39	0.40	0.53
Confectionery	0.70	0.60	0.47	0.55	0.43	0.51	0.56	0.49	0.21	0.25	0.37	0.45
Green tea	0.69	0.61	0.56	0.59	0.59	0.64	0.74	0.73	0.56	0.62	0.63	0.69
Coffee	0.78	0.77	0.51	0.55	0.57	0.60	0.80	0.67	0.52	0.55	0.52	0.56
Alcoholic beverages	0.88	0.86	0.60	0.63	0.58	0.61	0.89	0.84	0.72	0.77	0.62	0.66
Soybean paste	0.73	0.72	0.54	0.61	0.55	0.61	0.67	0.64	0.63	0.72	0.64	0.72
Median	0.70	0.61	0.44	0.51	0.48	0.55	0.69	0.66	0.39	0.47	0.42	0.52
Minimum	0.43	0.38	0.11	0.17	0.17	0.26	0.45	0.45	0.17	0.23	0.21	0.24
Maximum	0.88	0.86	0.71	0.76	0.76	0.81	0.89	0.84	0.72	0.77	0.64	0.72

The residual method was performed to adjust the energy intake. FFQ: 47-item short food frequency questionnaire, DR: four non-consecutive 3-day weighed dietary records at intervals of 3 months (12 days), SR: Spearman's rank correlation coefficient

De-attenuated SR = observed SR × (1 + λx/n)^0.5, where λx is the ratio of within- to between-individual variance for food group intake, and n is the number of dietary surveys = 4. Rank of food group intake was transformed using the probit function (ref. [21])

**Table 4:** Comparison of two FFQs with 12-day-weighed food record and FFQ1 with FFQ2 for energy-adjusted food-group intakes, based on cross-classification by quintile (%) in men and women

				Men (*n* = 143)									Women (*n* = 145)					
	FFQ1 vs FFQ2			DR vs FFQ1			DR vs FFQ2			FFQ1 vs FFQ2			DR vs FFQ1			DR vs FFQ2		
	Same category	Same and	Extremecategory	Same category	Same and	Extremecategory	Same category	Same and	Extremecategory	Same category	Same and	Extremecategory	Same category	Same and	Extremecategory	Same category	Same and	Extremecategory
		adjacent			adjacent			adjacent			adjacent			adjacent			adjacent	
		category			category			category			category			category			category	
Rice	41	90	0	35	75	0	35	78	2	43	86	0	37	76	1	39	77	1
Bread	45	87	1	40	86	1	41	83	1	48	89	0	39	83	1	40	82	1
Noodles	42	78	1	31	71	1	31	73	1	39	81	0	28	61	5	33	71	2
Potatoes	32	70	3	23	58	6	20	56	5	38	72	0	23	64	2	28	62	3
Soybean products	43	85	1	29	59	1	25	66	2	46	79	1	28	65	2	30	66	3
Green vegetables	41	76	1	25	65	2	28	66	3	36	80	1	22	67	3	34	67	3
Other vegetables	34	71	2	28	64	3	20	61	6	41	74	1	21	57	4	26	59	6
Fruit	49	83	1	39	73	2	38	78	1	38	83	1	31	72	2	34	72	3
Mushrooms	36	75	3	27	59	6	32	64	6	41	79	1	23	65	3	31	60	6
Seaweeds	31	71	4	28	66	6	22	69	6	32	70	3	19	61	7	27	63	6
Fish	35	80	1	28	59	4	36	73	2	49	81	1	34	68	0	31	72	0
Meat	42	78	3	22	59	2	27	63	3	40	80	1	27	65	3	27	64	5
Eggs	50	87	0	29	65	1	30	63	2	50	79	0	27	65	3	30	72	3
Milk	51	90	1	43	85	0	50	87	0	61	92	0	39	75	1	39	79	1
Oils	36	76	1	23	63	4	26	67	3	35	75	1	27	63	5	35	68	6
Confectionery	42	81	1	28	67	1	29	63	2	34	69	3	28	60	8	30	66	3
Green tea	51	85	1	39	78	1	48	83	1	54	86	1	39	80	1	41	79	1
Coffee	45	90	1	34	72	1	36	76	1	62	90	1	32	70	2	36	77	1
Alcoholic beverages	55	94	0	39	78	0	34	78	0	54	90	0	46	86	1	39	81	1
Soybean paste	45	86	1	40	76	1	40	75	1	43	81	2	35	77	1	40	81	1
Median	42	81	1	29	67	1	31	69	2	41	80	1	28	65	2	33	71	3
Range	(31–55)	(70–94)	(0–4)	(22–43)	(58–86)	(0–6)	(20–50)	(56–87)	(0–6)	(32–62)	(69–92)	(0–3)	(19–46)	(57–86)	(0–8)	(26–41)	(59–82)	(0–6)

The residual method was performed to adjust the energy intake. FFQ: 47-item short food frequency questionnaire, DR: four non-consecutive 3-day weighed dietary records at intervals of 3 months (12 days)

### Correlation coefficient and agreement rate for validity

For validity in men, energy-adjusted SRs for FFQ1 vs. DRs were distributed from 0.11 (potatoes) to 0.71 (milk), with a median of 0.44. De-attenuated SRs were distributed from 0.17 (potatoes) to 0.76 (bread and milk), with a median of 0.51 (Table 3). In women, energy-adjusted SRs for FFQ1 vs. DRs were distributed from 0.17 (seaweeds) to 0.72 (alcoholic beverages), with a median of 0.39. De-attenuated SRs were distributed from 0.23 (seaweeds) to 0.77 (alcoholic beverages), with a median of 0.47 (Table 3). The SRs by FFQ1 or FFQ2 vs. DRs can be indices for validity. For both the energy-adjusted SRs and the de-attenuated SRs, the median correlation coefficients by the FFQ2 were 0.04 higher than those by the FFQ1 for men. For women, the median of energy-adjusted SR by FFQ2 was 0.03 higher than that of FFQ1 and the median of de-attenuated SR was 0.05 higher. When miso was included as a soy product, the de-attenuated SRs were 0.61 (FFQ1 vs. DR) and 0.61 (FFQ2 vs. DR) for men, and 0.47 (FFQ1 vs. DR) and 0.52 (FFQ2 vs. DR) for women.

The median agreement rates between the FFQ1 and DRs in men and women were 29% and 28%, respectively (Table 4). The median agreement rates for the same and adjacent categories were 67% (range 58% (potatoes) to 86% (bread)) for men and 65% (range 57% (other vegetables) to 86% (alcoholic beverages)) for women. The agreement rates between the FFQ2 and DRs showed almost the same median value in men and women. The agreement rates for six food groups for men (rice, bread, milk, green tea, alcoholic beverages, and soybean paste) and six food groups for women (rice, bread, milk, green tea, alcoholic beverages, and soybean paste) were greater than or equal to 75% for both the FFQ1 and FFQ2. Extreme disagreement rates (median) for FFQ1 in men and women were 1 and 2%, respectively.

### The assessment of food intake range by Bland–Altman plot graphs

Bland–Altman plot graphs for the consumption of rice (a), fish (b), meat (c), milk (d), other vegetables (e), and fruit (f) among men and women are shown in Additional file 3. Rice (a) showed a wide distribution on the *x* axis for men; positive and negative errors occurred randomly in individuals with intermediate mean consumption (200–500 g/day). Fish (b) and meat (c) were widely distributed in terms of average intake (*x*-axis), and the difference, which was FFQ1 − DR (*y*-axis), tended to become negative as the intake increased. Due to differences in the average reflecting − 34 g (fish) and − 67 g (meat) for men, these food groups were underestimated in the FFQ1. Regarding milk intake (d), the mean intake in the FFQ1 and DRs was concentrated close to the intersection of the *x*- and *y*-axes for both men and women. For individuals with low mean levels of consumption, the differences between the errors in the FFQ1 and DRs tended to be less on the *y*-axis. Greater underestimation was observed in men than in women for the intake of other vegetables (e) and fruit (f) on the FFQ1.

## Discussion

The results presented in this study using a 47-item short FFQ developed in the central area of Japan demonstrated high reproducibility and reasonable validity for many food groups over a wide area of Japan. For reproducibility, the median energy-adjusted SRs between the FFQ1 and FFQ2 were 0.61 and 0.66 for men and women, respectively. For food groups, the differences between the FFQ1 and FFQ2 were negligible because robust Z scores were within the acceptable range. For validity, the median de-attenuated SRs between the FFQ1 and DR were 0.51 for men and 0.47 for women. The agreement rates based on cross-classification by quintile were comparable to those in a previous study [25]. The agreement rates for validity between the FFQs and DRs were also reasonably acceptable. Few extreme misclassifications were found using this FFQ.

### Absolute dietary intake estimated by FFQ

Based on the robust Z scores, the amounts of food group intake were relatively underestimated in this 47-item short FFQ compared with the DRs. Previous research has reported that most FFQs with over 100 items often overestimate the absolute dietary intake because the reported amount of foods will increase overall when many items are asked [26, 27]. This 47-item short FFQ consists of 20 food groups, 11 of which contain only one food item; thus, the small underestimation should be acceptable. In addition, judging from the robust Z scores and the Bland–Altman plot graphs, the estimated intakes on the FFQ1 were severely underestimated for meat for men and for other vegetables for both men and women.

### Reproducibility of intake by food groups

A previous study that evaluated 15 food groups using the 47-item FFQ has already confirmed its reproducibility in the central area of Japan (Aichi Prefecture) [12]. That study showed that energy-adjusted SRs were 0.65 as the median (range 0.59–0.80) for 844 men and 0.60 (range 0.56–0.69) for 1074 women. Although the minimum SRs were lower than those in previous reports in both men and women, the present results showed similar SRs. Thus, the reproducibility of the 47-item FFQ is considered generalizable throughout Japan.

Regarding short FFQs (40–66 items) developed in Japan—Ogawa (40 questions) [28], Date (40 questions) [29], Maruyama (55 questions) [30], Kobayashi (58 questions) [31], and Yokoyama (66 questions) [25]—the median SRs ranged from 0.50 to 0.60 in Ogawa [28], Date [29], and Maruyama [30]. Because the median SRs of the 47-item short FFQ were 0.61 for men and 0.66 for women, the reproducibility of the weight by food group was slightly better in the present study. This short FFQ was developed using multiple regression analysis (MRA) of 102 items from a semiquantitative FFQ [32]. The food list on the 102-item semiquantitative FFQ included foods with a high supply rate of 21 nutrients and energy by contribution analysis (CA). The MRA is based on the variance of nutrient intake. The cumulative R^2^ estimated by MRA can generally be explained by a smaller number of foods compared with the cumulative percent CA. Additionally, because the foods listed in short FFQs are commonly consumed and easy to recognize, the 47-item FFQ might have higher reproducibility.

### Validity of intake by food groups

We assessed the validity of intake by 20 food groups estimated by the present 47-item short FFQ. The number of food groups in previous studies involving short FFQs developed in Japan ranged from 10 to 30 [25, 28, 30, 31]. Generally, the SR for validity was higher when the number of food groups was small, with medians ranging from 0.51 to 0.60 in 10 food groups (Ogawa [28], Maruyama [30]) and from 0.44 to 0.48 in over 30 food groups (Kobayashi [31], Yokoyama [25]). The validity of current short FFQs is comparable to that of latter SRs. In addition, when the participants’ intakes were classified into quintiles, the evaluation based on agreement rates showed that the medians were at the same levels as those in previous studies (Yokoyama 70% vs. our FFQ2 69% for men, and Yokoyama 64% vs. our FFQ2 69% for women). Therefore, our FFQ is considered to be reasonably valid.

### Characteristics of food groups with high or low reproducibility and validity

The reproducibility and validity of staple foods were relatively higher, especially for rice and bread; milk and alcoholic beverages also showed higher reproducibility and validity for men and women. However, caution is needed when interpreting the intake of some food groups with relatively low SRs. For men, the reproducibility and validity of potatoes were considerably low. Although the reproducibility of mushrooms was acceptable, the validity with both the de-attenuated SR and the cross-classification rate was relatively low. The intakes of meat and other vegetables in men were underestimated, but the categorization power was sustained. For women, the validity of other vegetables, mushrooms, seaweeds, and confectionery were relatively low with de-attenuated SRs in the 0.20s. Especially for seaweeds, the SRs for reproducibility were also low for women. The intake of other vegetables in women was underestimated. In previous FFQs developed in Japan, similar findings were also observed for these food groups [2, 12, 33]. Several reasons could explain these findings. First, it may be easy to observe between-person variation in drinks because drink intakes are widely distributed. However, the ranges of portion sizes were very small (1.0–3.0 g) for dried foods (e.g., seaweeds, dried mushrooms). In addition, because the amount of dried foods and added water can be a systematic error in dietary assessment studies, the correlation coefficient may be underestimated. Another reason is the recognition and memory of individual dietary intakes. In other words, drinks are easier to remember because they are taken alone, while foods are often consumed as a mixture, which makes it more difficult to remember their frequency. Staple foods are also easy to remember as a single dish. In addition, regarding Japanese dietary habits, those who consume bread instead of rice as a staple food are likely to drink milk at the same time [34], which would lead to high reproducibility and validity for both milk and bread [31].

The classification of food groups resulted in 15 groups in a previous reproducibility study [12]. In the present study, this has been expanded to 20 groups based on the Standard Tables of Food Composition in Japan (seventh revised edition). Miso (soybean paste) was classified as a soybean product in the past edition, but this was changed to a seasoning in the revised edition; therefore, soybean products and miso were evaluated separately in this study. By increasing the number of food groups to 20, it was possible to compare the present FFQs with other FFQs and evaluate the relationship between foods and diseases in more detail.

### Usefulness of the FFQ1 and FFQ2 in the analysis of validity study

Whether DRs should be compared with the FFQ1 or FFQ2 when designing a validation study remains controversial [1, 35]. Our previous studies on the same FFQ used the FFQ1 for validation [10, 12]. The present study found that the median de-attenuated SRs between the FFQ2 and DRs were slightly higher than those for the FFQ1. According to Willett [1], the correlation coefficient between the FFQ1 and DRs often underestimates the *true* correlation, whereas that between the FFQ2 and DRs provides an *optimistic* correlation. Since the FFQ2 was administered after the DR survey, the participants may have been able to provide the *real* frequency. Previous validity studies by Yokoyama [25], Ogawa [28], Date [29], and Willett [36] treated the FFQ2 as a comparison, and Maruyama [30] assessed validity through a comparison between the FFQ1 and FFQ3 (the third administration of FFQ). Kobayashi [31] used the average value from the FFQ1 and four FFQs. It is difficult to conclude which value of the FFQ is valid for evaluation, as previous studies have used a variety of methods. However, the results from this study for both the FFQ1 and FFQ2 may provide important evidence for future research on more appropriate validation methods.

### Limitations of the study

In this study, we recruited a sufficient number of participants throughout Japan; however, some limitations should be noted. First, although we defined food group intake as the usual dietary intake based on 12-day DRs, this did not reflect the actual daily food intake. According to Fukumoto et al. [37], the number of days required to assess the mean intake of nutrients with 95% confidence intervals within 5% deviation of an individual’s mean from the usual (“true”) intake using DR method is 2–4 weeks for energy, carbohydrates, and protein, and 7–40 weeks for fat, vitamins, and minerals. Since within-individual variations for food group intake are generally larger than those for nutrition intake [38], the 12-day DRs used in the present study may have been rather short. However, longer DRs could increase the burden on participants and result in more dropouts, leading to selection bias. Therefore, we prioritized the feasibility of dietary surveys over the number of survey days statistically required. Second, the influence of selection bias should be noted because the volunteer population participating in a year-long DR survey will be more health conscious than the general Japanese population. Third, SSBs were not included in the short FFQ because the ability of the 47-item FFQ to estimate nutrients semiquantitatively is limited. The effects of SSB consumption on cardiovascular disease (CVD) morbidity and mortality and risk factors have been reported [39, 40]. The effects on CVD incidence and risk factors have also been studied in Japanese populations, but further studies including mortality risk would be needed to accumulate evidence [41, 42]. Fourth, although we evaluated the reproducibility and validity of the FFQ developed in Aichi prefecture over a wide area of Japan, we were unable to evaluate the reproducibility and validity in each area. Thus, we may need to examine between-region differences in the reproducibility and validity of this FFQ. Finally, our survey was set to cover a non-consecutive 3-day period, but some high-calorie foods, such as cakes and sweetbreads, may not have been included in the usual daily diet, as these foods are often consumed only on special occasions (e.g., birthdays, parties); therefore, some participants may have underreported these foods.

## Conclusion

The present study assessed the reproducibility and validity of the short FFQ for food group intake in multiple populations representing all of Japan. Both the FFQ1 and FFQ2 showed higher reproducibility and reasonable validity. Therefore, this short FFQ is considered suitable for the assessment of dietary intake in cohort studies involving middle-aged Japanese populations.

## Supplementary Information

**Additional file 1.** Design of reproducibility and validity study for a 47-item short FFQ.

**Additional file 2.** Spearman's rank correlation coefficients of energy-adjusted intakes (density method) for reproducibility and validity for men and women.

**Additional file 3.** Bland Altman plot graphs for the consumption of rice (a), fish (b), meat (c), milk (d), other vegetables (e), and fruit (f) among men and women.

## Data Availability

The data sets generated and analyzed in the present study are not publicly available because we did not obtain informed consent from the participants for the open use of individual data.
