# Supplementary material for: Reproducibility and validity of food group intake in a short food frequency questionnaire for the middle-aged Japanese population
Source: Environ Health Prev Med. 2021 Mar 2;26:28. doi: 10.1186/s12199-021-00951-3 (PMC7923820; doi:10.1186/s12199-021-00951-3)
Supplement: Supplementary file 1 — Additional file 1. Design of reproducibility and validity study for a 47-item short FFQ. [file 12199_2021_951_MOESM1_ESM.pptx]

## Slide 1
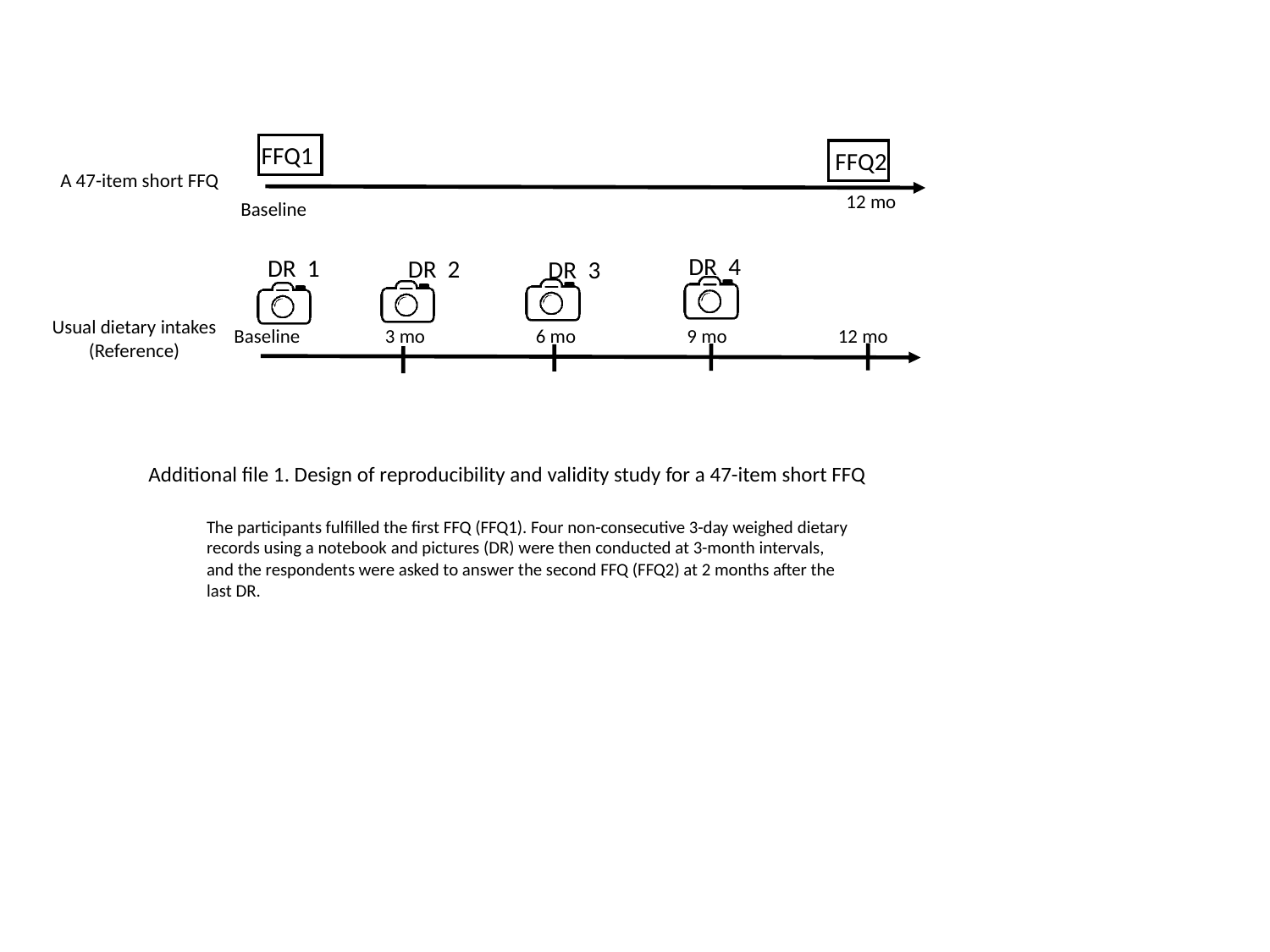

FFQ1
 FFQ2
A 47-item short FFQ
12 mo
Baseline
DR 4
DR 1
DR 2
DR 3
Usual dietary intakes (Reference)
Baseline
3 mo
6 mo
9 mo
12 mo
Additional file 1. Design of reproducibility and validity study for a 47-item short FFQ
The participants fulfilled the first FFQ (FFQ1). Four non-consecutive 3-day weighed dietary records using a notebook and pictures (DR) were then conducted at 3-month intervals, and the respondents were asked to answer the second FFQ (FFQ2) at 2 months after the last DR.
