# Supplementary material for: Reproducibility and validity of food group intake in a short food frequency questionnaire for the middle-aged Japanese population
Source: Environ Health Prev Med. 2021 Mar 2;26:28. doi: 10.1186/s12199-021-00951-3 (PMC7923820; doi:10.1186/s12199-021-00951-3)
Supplement: Supplementary file 3 — Additional file 3. Bland Altman plot graphs for the consumption of rice (a), fish (b), meat (c), milk (d), other vegetables (e), and fruit (f) among men and women. [file 12199_2021_951_MOESM3_ESM.pdf]

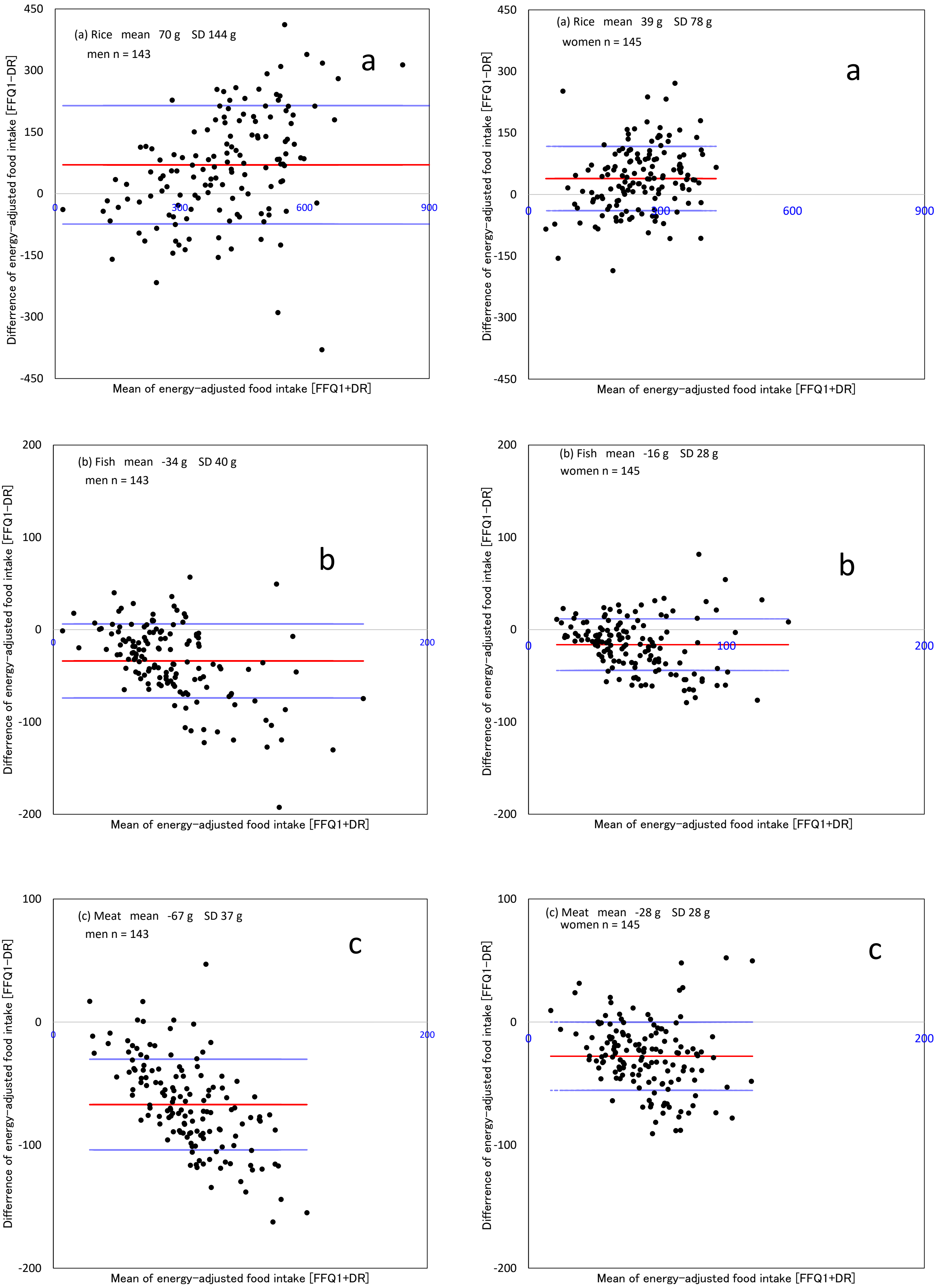
(a) Rice mean 70 g SD 144 g
men n = 143
a
Differrence of energy-adjusted food intake [FFQ1−DR]
Mean of energy-adjusted food intake [FFQ1+DR]
450
300
150
0
-150
-300
-450
0
300
600
900
(a) Rice mean 39 g SD 78 g
women n = 145
a
Differrence of energy-adjusted food intake [FFQ1−DR]
Mean of energy-adjusted food intake [FFQ1+DR]
(b) Fish mean -34 g SD 40 g
men n = 143
b
Differrence of energy-adjusted food intake [FFQ1−DR]
Mean of energy-adjusted food intake [FFQ1+DR]
200
100
0
-100
-200
(b) Fish mean -16 g SD 28 g
women n = 145
b
Differrence of energy-adjusted food intake [FFQ1−DR]
Mean of energy-adjusted food intake [FFQ1+DR]
(c) Meat mean -67 g SD 37 g
men n = 143
c
Differrence of energy-adjusted food intake [FFQ1−DR]
Mean of energy-adjusted food intake [FFQ1+DR]
(c) Meat mean -28 g SD 28 g
women n = 145
c
Differrence of energy-adjusted food intake [FFQ1−DR]
Mean of energy-adjusted food intake [FFQ1+DR]

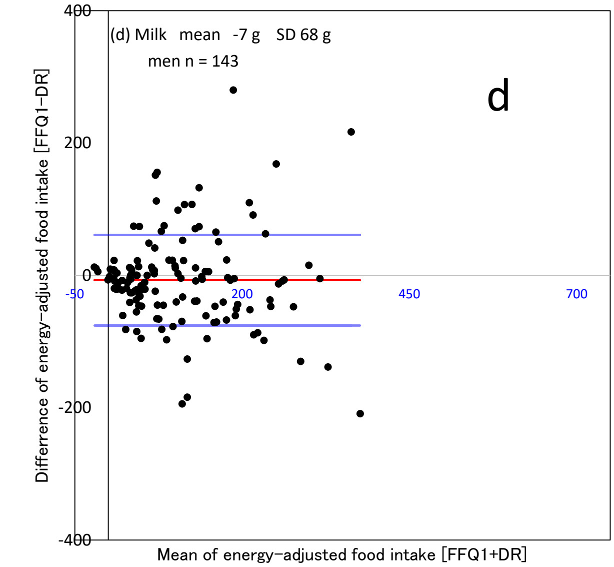
(d) Milk mean -7 g SD 68 g
men n = 143
d
Differrence of energy−adjusted food intake [FFQ1−DR]
400
200
0
-200
-400
-50
200
450
700
Mean of energy−adjusted food intake [FFQ1+DR]

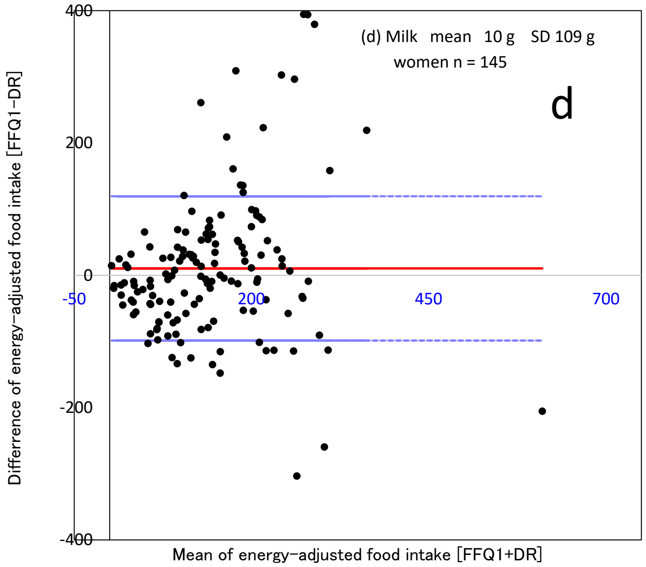
(d) Milk mean 10 g SD 109 g
women n = 145
d
Differrence of energy−adjusted food intake [FFQ1−DR]
400
200
0
-200
-400
-50
200
450
700
Mean of energy−adjusted food intake [FFQ1+DR]

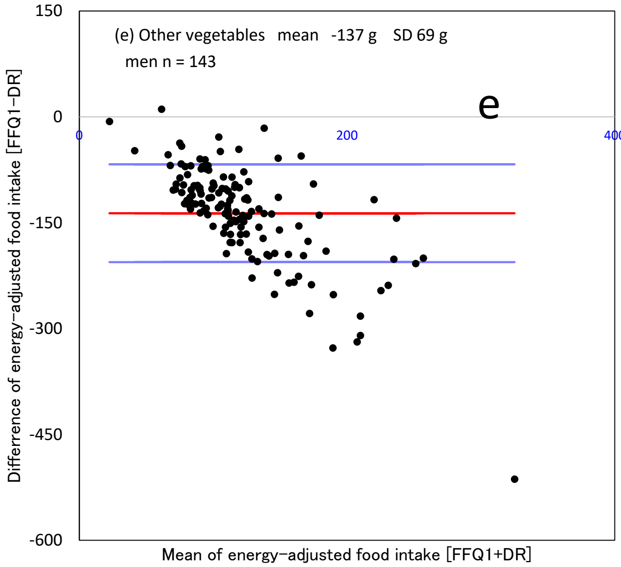
(e) Other vegetables mean -137 g SD 69 g
men n = 143
e
Differrence of energy−adjusted food intake [FFQ1−DR]
150
0
-150
-300
-450
-600
0
200
400
Mean of energy−adjusted food intake [FFQ1+DR]

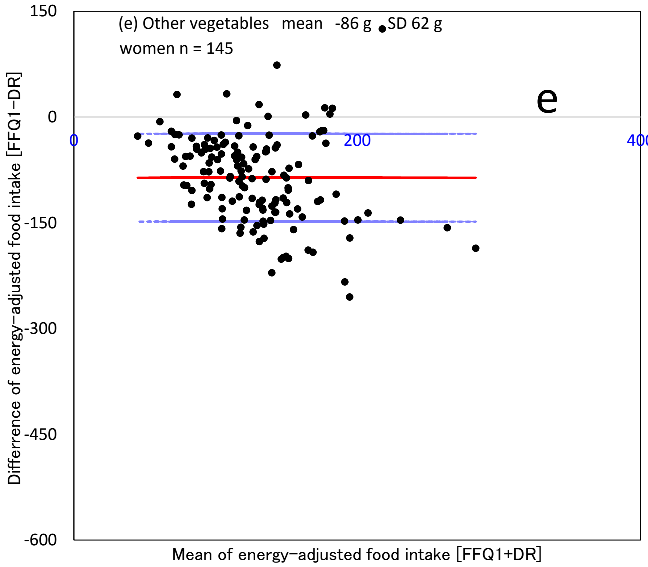
(e) Other vegetables mean -86 g SD 62 g
women n = 145
e
Differrence of energy−adjusted food intake [FFQ1−DR]
150
0
-150
-300
-450
-600
0
200
400
Mean of energy−adjusted food intake [FFQ1+DR]

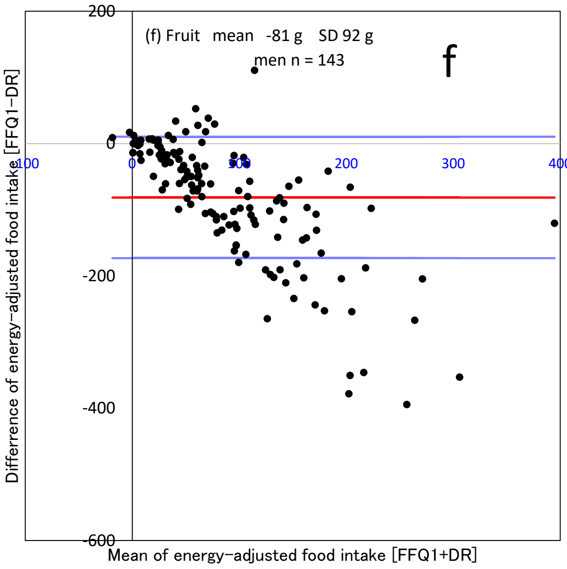
(f) Fruit mean -81 g SD 92 g
men n = 143
f
Differrence of energy−adjusted food intake [FFQ1−DR]
200
0
-200
-400
-600
-100
0
100
200
300
400
Mean of energy−adjusted food intake [FFQ1+DR]

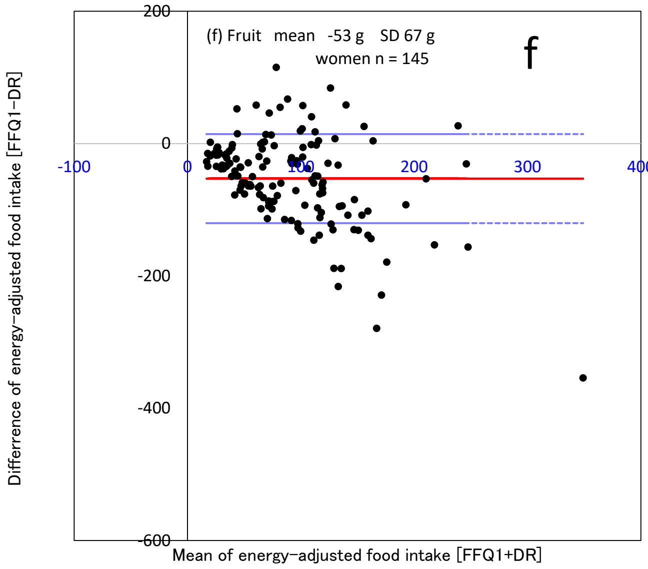
(f) Fruit mean -53 g SD 67 g
women n = 145
f
Differrence of energy−adjusted food intake [FFQ1−DR]
200
0
-200
-400
-600
-100
0
100
200
300
400
Mean of energy−adjusted food intake [FFQ1+DR]

Footnote: rice (a), fish (b), meat (c), milk (d), other vegetables (e), and fruit (f), left men, right: women
Red line: mean, blue line: +/- SD

Additional file 3: Bland Altman plot graphs for the consumption of rice (a), fish (b), meat (c), milk (d) , other vegetables (e), and fruit (f) among men and women
